# Association Between Concussions and Suicidality in High School Students in the United States

**DOI:** 10.3389/fneur.2022.810361

**Published:** 2022-04-12

**Authors:** Grant L. Iverson, Justin E. Karr

**Affiliations:** ^1^Department of Physical Medicine and Rehabilitation, Harvard Medical School, Boston, MA, United States; ^2^Spaulding Rehabilitation Hospital, Charlestown, MA, United States; ^3^Spaulding Research Institute, Charlestown, MA, United States; ^4^MassGeneral Hospital for Children Sports Concussion Program, Boston, MA, United States; ^5^Home Base, A Red Sox Foundation and Massachusetts General Hospital Program, Charlestown, MA, United States; ^6^Department of Psychology, University of Kentucky, Lexington, KY, United States

**Keywords:** suicide, concussion, head injury, traumatic brain injury, sports

## Abstract

**Importance:**

Prior research has shown a statistically significant association between sustaining a concussion and suicidality in adolescents, but this prior research controlled for relatively few variables predictive of suicidality.

**Objective:**

To examine whether sustaining a concussion remained a significant predictor of suicidality after controlling for relevant covariates (e.g., sexual abuse/assault, bullying, substance use, depression), hypothesizing that the relationship between concussion and suicidality would become non-significant after controlling for these variables.

**Design:**

This study involved secondary data analysis of the 2019 Youth Risk Behavior Surveillance (YRBS) System, a national cross-sectional study of adolescents. Analyses were stratified by gender.

**Setting:**

A national sampling of U.S. high school students.

**Participants:**

Eleven thousand two hundred sixty-two students in the YRBS database, including 5,483 boys and 5,779 girls.

**Exposure(s):**

Participants included in the analyses reported whether, in the last year, they experienced a concussion and/or suicidality.

**Main Outcomes and Measures:**

The main outcome was suicidality (i.e., ideation, planning, attempt), which was predicted by concussion in an unadjusted analysis and by concussion along with other risk factors in a multivariable analysis.

**Results:**

The final sample included 11,262 participants with available data on concussion and suicidality in the last year (14–18 years-old; 51.3% girls; 49.0% White). Per unadjusted odds ratios with 95% confidence intervals, there was a relationship between concussion and suicidal ideation [girls: OR = 1.46 (1.24, 1.73); boys: OR = 1.69 (1.41, 2.03)], planning (girls: OR = 1.39 [1.16, 1.66]; boys: OR = 1.76 [1.44, 2.14]), and attempt [girls: OR = 1.70 (1.32, 2.19); boys: OR = 3.13, (2.37, 4.15)]. These relationships became mostly non-significant after controlling for relevant risk factors for suicidality. The adjusted odds ratios showed no relationship between concussion and suicidal ideation [girls: OR = 1.11 (0.86, 1.44); boys: OR = 1.24 (0.92, 1.69)] or planning (girls: OR = 1.07 [0.82, 1.40]; boys: OR = 1.12 [0.82, 1.55]); but a significant relationship with suicide attempts in boys [OR = 1.98 (1.28, 3.04)], but not girls [OR = 1.05 (0.74, 1.49)].

**Conclusions and Relevance:**

There was an association between concussion and suicidality in U.S. high school students; however, after controlling for other variables (e.g., depression, sexual abuse/assault, illicit drug use), there was no association between concussion and suicidality aside from a significant relationship between concussion and attempts in boys.

## Introduction

Suicidal thoughts and behaviors during adolescence are considered to be a public health problem, and many factors are known to be associated with increased risk for suicidality including genetics (1); female sex (2–4); mental disorders (5), such as depression (5, 6) and anxiety (6); impulsivity (7); attention-deficit hyperactivity disorder (8, 9); chronic illness (10); and sleep problems (11, 12). Substance use is associated with suicidality in adolescents (10), including illicit drugs (13, 14), marijuana (14, 15), alcohol (13, 15), and tobacco (13, 15). Psychosocial stressors and adverse childhood experiences also are associated with suicidality, including cyberbullying (16–18); not feeling safe at school (19); and sexual abuse, physical abuse, emotional abuse, and neglect (20–23).

Parental psychological distress (24), marital problems (25, 26), academic problems (27), and being bullied (27, 28) at school are risk factors for mental health difficulties in youth. In contrast, a high level of physical activity, and participation in team sports, are associated with higher self-esteem and life satisfaction, and lower risk for psychological distress (29–32). Adolescents who are physically active have a lower risk for experiencing a mood disorder (33) and suicidality (34). Adolescents who participate in sports are less likely to report depression (35) or suicidal ideation (35–37). Involvement in school sports during adolescence is associated with better mental health in young adulthood (38), and adolescents who are physically active in general, and maintain that behavior into early adulthood, experience better mental health (39). Factors that are associated with *lower* risk for suicidality in youth include regular exercise (40) and if they perceive that their parents understand their problems, monitor their academic and leisure time activities, and respect their privacy (3).

In recent years, epidemiological survey studies have found an association between sustaining a concussion, mostly during sports or recreational activities, and suicidality in high school students (41–43)—even after controlling for some factors known to be associated with suicidality, such as sex (2, 42, 43), depression (41), and bullying (42). However, none of these studies controlled for sexual abuse or assault, and none of the studies statistically modeled a more comprehensive set of factors known to be associated with suicidality in youth. Examining these associations is complex because some associations are small, and depression can be both a direct risk factor (i.e., a determinant) and a mediating variable (e.g., bullying leads to depression and suicidality). This gap in the literature was addressed, in the present study, by examining data from the 2019 “Youth Risk Behavior Survey” (YRBS) (44). Every 2 years, the Center for Disease Control and Prevention in the United States conducts a national survey of high school students (i.e., the YRBS). The purpose of this study is to examine the association between concussion and suicidality in high school students in the United States. It was hypothesized that in univariate analyses concussion would be associated with suicidality, similar to prior studies, but when controlling for other variables the association would be greatly attenuated or become non-significant.

## Materials and Methods

### Survey Methodology

The national survey is conducted every 2 years, during odd-numbered years, among students in grades 9–12 who attend a public and private schools in the United States. The Institutional Review Board of the CDC approved the protocol for the survey. The survey procedures were designed to allow for voluntary and anonymous participation. The students completed the survey in school during a single class period (~45 min).

The total national sample included 13,872 students from 136 schools (44). The response rate for the schools was 75.1%, the response rate for the students was 80.3%, yielding an overall response rate of 60.3% [i.e., (student response rate) × (school response rate)]. A questionnaire failed quality control if fewer than 20 responses remained after editing or if the student marked the same answer for 15 or more consecutive questions—yielding 195 questionnaires that failed quality control. The survey data from 1991 through 2019 are publicly available (https://www.cdc.gov/healthyyouth/data/yrbs/).

### Survey Questions and Combined Variables

There were 99 questions, 89 of which were included in the standard questionnaire and 10 additional questions were added to address areas of interest for CDC and other stakeholders. The survey questions, recall periods, response options, and definitions of each variable are provided in the 2019 YRBS questionnaire and data user's guide available on the website (https://www.cdc.gov/healthyyouth/data/yrbs/). The survey questions used in this study are reprinted in Table 1. In addition, variables were combined based on two or more survey questions, and those variables are also presented in Table 1.

**Table 1:** Survey questions and combined variables used in this study.

Q15. During the past 30 days, on how many days did you not go to school because you felt you would be unsafe at school or on your way to or from school? Endorsing 1 or more was coded as “Felt Unsafe at School.”
Q16. During the past 12 months, how many times has someone threatened or injured you with a weapon such as a gun, knife, or club on school property? Endorsing 1 or more was coded as “Threatened on School Property.”
Q19. Have you ever been physically forced to have sexual intercourse when you did not want to? Endorsing this question as “yes” was coded as “Sexual Abuse/Assault.”
QN17. During the past 12 months, how many times were you in a physical fight? Coded “yes” if endorsed as 1 or more.
QN20. During the past 12 months, how many times did anyone force you to do sexual things that you did not want to do? (Count such things as kissing, touching, or being physically forced to have sexual intercourse.)
QN21. During the past 12 months, how many times did someone you were dating or going out with force you to do sexual things that you did not want to do? (Count such things as kissing, touching, or being physically forced to have sexual intercourse.) (Endorsed as yes if the person had dated and this happened 1 or more times)
QN22: During the past 12 months, how many times did someone you were dating or going out with physically hurt you on purpose? (Count such things as being hit, slammed into something, or injured with an object or weapon.) (Endorsed as yes if the person had dated and this happened 1 or more times)
Q23. During the past 12 months, have you ever been bullied on school property?
Q24. During the past 12 months, have you ever been electronically bullied? (Count being bullied through texting, Instagram, Facebook, or other social media.) Endorsing either Q23 or Q24 as “yes” was coded as “Bullied.”
Q25. During the past 12 months, did you ever feel so sad or hopeless almost every day for two weeks or more in a row that you stopped doing some usual activities? Endorsing this question as “yes” was coded as “Depression in Past Year.”
Q26. During the past 12 months, did you ever seriously consider attempting suicide?
Q27. During the past 12 months, did you make a plan about how you would attempt suicide?
QN28. During the past 12 months, how many times did you actually attempt suicide? This variable was coded as “yes” if the student reported one or more attempts. Coded “yes” if 1 or more.
QN32. During the past 30 days, on how many days did you smoke cigarettes? Coded yes if 1 or more.
QN42. During the past 30 days, on how many days did you have 4 or more drinks of alcohol in a row, that is, within a couple of hours (if you are female) or 5 or more drinks of alcohol in a row, that is, within a couple of hours (if you are male)? Coded as “yes” if 1 or more for the variable “Current Binge Drinking.”
During your life, how many times have you (i) taken prescription pain medicine without a doctor's prescription or differently than how a doctor told you to use it (Q49); (ii) used any form of cocaine, including powder, crack, or freebase (Q50); (iii) sniffed glue, breathed the contents of aerosol spray cans, or inhaled any paints or sprays to get high (Q51); (iv) used heroin (also called smack, junk, or China White) (Q52); (v) used methamphetamines (also called speed, crystal meth, crank, ice, or meth) (Q53); (vi) used ecstasy (also called MDMA or Molly) (Q54); or (vii) used a needle to inject any illegal drug into your body (Q56)? The QN variables were used for “yes” or “no” and the regular variables were used to classify as 3 or more times.
Used Illicit Drugs 3 or More Times: Reported using one or more of the following drugs 3 or more times during lifetime: (i) prescription pain medication with a prescription; (ii) cocaine; (iii) inhalants; (iv) heroin; (v) methamphetamines; (vi) ecstasy/MDMA; or (vii) used a needle to inject any illegal drug into your body.
Q78. During the past 7 days, on how many days were you physically active for a total of at least 60 min per day? (Add up all the time you spent in any kind of physical activity that increased your heart rate and made you breathe hard some of the time.) Endorsing 5 or more days was coded as “Physically Active 5+ Days.”.
QN82. During the past 12 months, on how many sports teams did you play? (Count any teams run by your school or community groups.) Coded as “yes” if 1 or more teams.
Q83. During the past 12 months, how many times did you have a concussion from playing a sport or being physically active? Endorsing one or more was coded as “yes.”
Q88. On an average school night, how many hours of sleep do you get? Those who endorsed 5 or fewer hours were coded as “Insufficient Sleep.”
Q89. During the past 12 months, how would you describe your grades in school? Those who endorsed “Mostly D's” or “Mostly F's” were classified as “Low Grades.”
Q90. During the past 30 days, how many times have you taken prescription pain medicine without a doctor's prescription or differently than how a doctor told you to use it? Endorsing 1 or more was coded as “yes” for “Opiate Use in Past 30 Days.”
No Adversity or Depression: Responded “no” to the following questions: felt unsafe at school; threatened on school property; being in a physical fight; ever forced to have sexual intercourse; forced sexual things in past year; forced sexual things while dating (if the person dated in the past year); being physically hurt by someone while dating (if the person dated in the past year); bullied on school property; bullied electronically; and being depressed in the past year.

*These questions were derived from the 2019 YRBS Data User's Guide (https://www.cdc.gov/healthyyouth/data/yrbs/)*.

### Statistical Analyses

The frequency of suicidal ideation, making a suicide plan, and making a suicide attempt was compared between boys and girls using χ^2^ tests, with *p* < 0.05 indicating a significant difference in frequency on these variables between gender groups. Three sets of binary logistic regressions were conducted separately in boys and girls, with (1) suicidal ideation, (2) making a suicide plan, and (3) making a suicide attempt serving as dichotomous dependent variables. Each suicide variable was predicted by each of the following variables in bivariate models, with the unadjusted odds ratio (OR) reported for each analysis: (1) being physically active for 5 or more days, (2) history of sexual abuse or assault, (3) feeling unsafe at school, (4) being threatened at school, (5) being bullied electronically or at school, (6) having on average D or F grades, (7) having insufficient sleep (i.e., 5 or fewer h on school nights), (8) current binge drinking, (9) current cigarette use, (10) lifetime use of 3 or more illicit drugs, (11) depression (i.e., feeling sad or hopeless), and (12) having a concussion from playing a sport or physical activity. Table 1 provides the specific question text of each of these variables. An OR above 1.00 with a 95% confidence interval (CI) not including 1.00 indicated the predictor was associated with greater odds of endorsing the dependent variable, whereas an OR below 1.00 with a 95% CI not including 1.00 indicated the predictor was associated with reduced odds of endorsing the dependent variable. A multivariable logistic regression was conducted including all predictors in the same model to examine the association between concussion and the suicide-related variables controlling for other variables protective against or increasing risk for suicide. The ORs can be interpreted in the same manner, but they reflect the increase or decrease in odds of suicidal ideation, suicide planning, and suicide attempt among adolescents with a concussion in the prior year, controlling for all other variables in the model.

## Results

There are 13,677 students in the database, of whom 151 had missing data on their sex and 72 had missing data on their age. Of those 13,442 students between the ages of 14 and 18, 13,243 (98.5%) answered the question about suicidal ideation, and of those there were 11,262 who answered the question about concussion as well. For the final sample of 11,262, there were 5,779 girls (51.3%) and 5,483 boys (48.7%). In regard to self-identified race and ethnicity, the sample had the following composition: 49.0% White, 15.8% Multiracial and Hispanic/Latinx, 13.6% Black/African American, 8.3% Hispanic/Latinx, 5.0% Asian, 4.8% Multiracial and non-Hispanic/Latinx, 0.8% American Indian or Alaskan Native, 0.4% Native Hawaiian or Pacific Islander, and 2.2% with no reported race/ethnicity.

**Figure 1:**
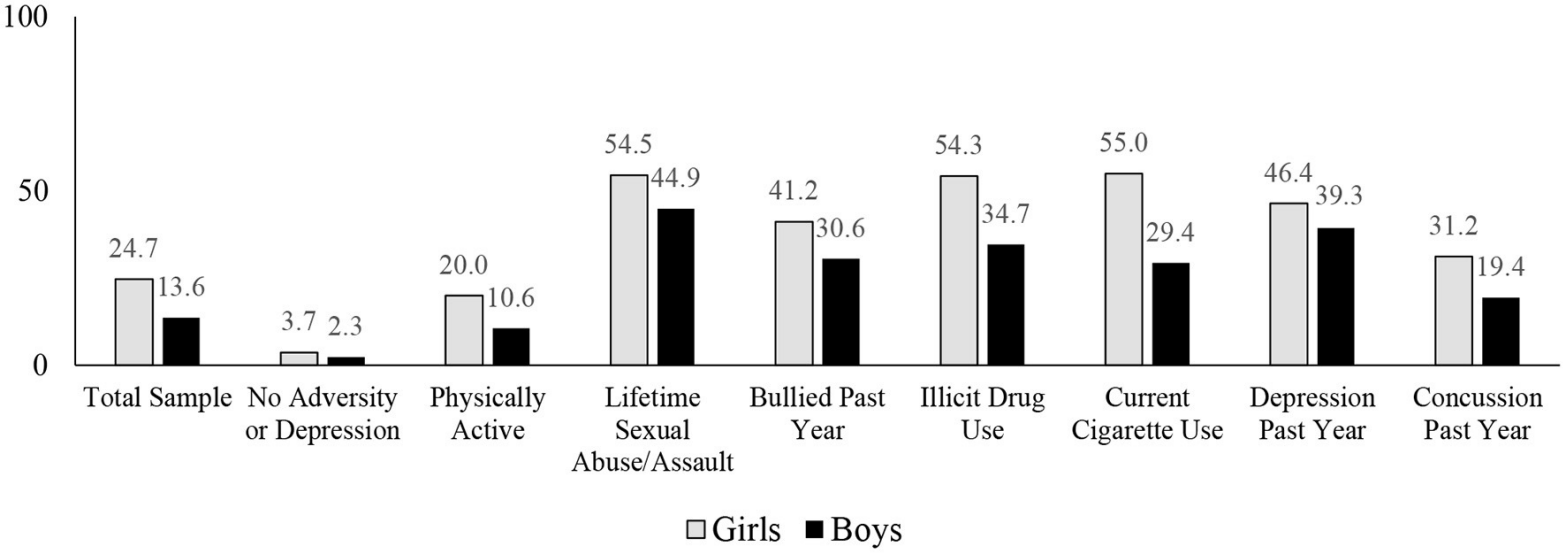
Percentages of high school students endorsing suicidal ideation over the past year. See Table 1 for the definitions of the variables. See Table 2 for the sample sizes. The no adversity or depression group responded “no” to all of the questions relating to sexual abuse or assault, feeling unsafe at school, being bullied, and being depressed in the past year. The physically active group reported being active for 60 min or more for at least 5 of the past 7 days. The illicit drug use groups reported a lifetime history of using drugs 3 or more times.

In the total sample, 19.3% endorsed having suicidal ideation in the past year, 15.7% endorsed having a plan for suicide, and 6.6% endorsed having attempted suicide. Girls endorsed suicidal ideation (24.7%) more frequently than boys [13.6%; χ^2^(1) = 221.49, *p* < 0.001; OR = 2.08 (95% CI: 1.89, 2.29)]; girls reported making a suicide plan (20.0%) more frequently than boys [11.3%; χ^2^(1) = 158.63, *p* < 0.001; OR = 1.96 (1.76, 2.18)]; and girls reported attempting suicide (11.4%) more frequently than boys [6.0%; χ^2^(1) = 75.63, *p* < 0.001; OR = 2.00 (95% CI: 1.71, 2.35)]. The percentages of youth endorsing suicidal ideation, plans, and attempts, stratified by sex and subgroups, are presented in Table 2. See Figure 1 for the percentages of youth, in specific subgroups, who endorsed suicidal ideation in the past year.

**Table 2:** Percentages of high school students endorsing suicidality.

**Group**	**Girls**						**Boys**						**Total**					
	**Suicidal ideation**		**Suicide plan**		**Suicide attempt**		**Suicidal ideation**		**Suicide plan**		**Suicide attempt**		**Suicidal ideation**		**Suicide plan**		**Suicide attempt**	
	*n*	%	*n*	%	*n*	%	*n*	%	*n*	%	*n*	%	*n*	%	*n*	%	*n*	%
Total sample	5,779	24.7%	5,751	20.0%	4,427	11.4%	5,483	13.6%	5,464	11.3%	4,074	6.0%	11,262	19.3%	11,215	15.8%	8,501	8.8%
Age 14	799	25.5%	792	21.5%	543	12.9%	604	13.7%	599	9.7%	403	5.2%	1,403	20.5%	1,391	16.4%	946	9.6%
Age 15	1,463	24.9%	1,459	20.2%	1,143	12.3%	1,372	12.8%	1,369	11.5%	985	5.8%	2,835	19.0%	2,828	16.0%	2,128	9.3%
Age 16	1,561	25.1%	1,555	20.2%	1,206	10.7%	1,473	14.3%	1,467	11.0%	1,087	6.6%	3,034	19.9%	3,022	15.7%	2,293	8.8%
Age 17	1,325	24.5%	1,316	19.4%	1,025	11.6%	1,307	13.1%	1,304	11.6%	1,022	5.2%	2,632	18.8%	2,620	15.5%	2,047	8.4%
Age 18+	631	22.3%	629	18.4%	510	8.6%	727	14.4%	725	12.4%	577	7.3%	1,358	18.1%	1,354	15.2%	1,087	7.9%
Ethnicity		–		–		–		–		–		–		–		–		–
Hispanic/Latinx	1,402	22.8%	1,395	20.0%	1,140	12.1%	1,311	12.4%	1,308	10.4%	1,007	6.1%	2,713	17.8%	2,703	15.4%	2,147	9.3%
Non-Hispanic/Latinx	4,330	25.3%	4,309	20.0%	3,258	11.1%	4,086	13.8%	4,073	11.6%	3,014	6.0%	8,416	19.7%	8,382	15.9%	6,272	8.6%
Race		–		–		–		–		–		–		–		–		–
White	2,852	24.6%	2,841	18.8%	2,295	9.5%	2,671	13.7%	2,667	11.4%	2,119	5.2%	5,523	19.3%	5,508	15.2%	4,414	7.4%
Black/African American	771	24.8%	765	21.0%	437	16.2%	758	10.7%	751	9.7%	414	8.2%	1,529	17.8%	1,516	15.4%	851	12.3%
Hispanic/Latinx	487	20.3%	483	16.8%	373	9.9%	446	11.0%	446	10.3%	316	5.7%	933	15.9%	929	13.7%	689	8.0%
Asian	283	22.6%	281	18.1%	220	6.8%	282	16.7%	282	14.2%	219	6.4%	565	19.6%	563	16.2%	439	6.6%
American Indian/Alaskan Native	40	40.0%	39	33.3%	27	18.5%	55	27.3%	55	18.2%	36	27.8%	95	32.6%	94	24.5%	63	23.8%
Multiracial and Hispanic/Latinx	914	24.1%	911	21.7%	767	13.2%	865	13.2%	862	10.4%	691	6.2%	1,779	18.8%	1,773	16.2%	1,458	9.9%
Multiracial and Non-Hispanic/Latinx	306	35.6%	306	27.5%	221	20.4%	238	19.3%	238	15.1%	174	5.2%	544	28.5%	544	22.1%	395	13.7%
Played on one or more sports teams	2,420	22.7%	2,412	18.9%	2,210	10.6%	2,507	11.8%	2,503	10.1%	2,213	5.6%	4,927	17.2%	4,915	14.4%	4,423	8.1%
Physically active 5+ days	2,072	20.0%	2,064	16.8%	1,639	9.1%	2,861	10.6%	2,856	8.9%	2,230	4.4%	4,933	14.5%	4,920	12.2%	3,869	6.4%
Sexual Abuse/Assault	492	54.5%	492	48.4%	441	37.2%	156	44.9%	156	39.7%	134	32.8%	648	52.2%	648	46.3%	575	36.2%
Bullied electronically or at school	1,729	41.2%	1,715	34.4%	1,348	21.5%	1,014	30.6%	1,008	25.0%	794	15.1%	2,743	37.3%	2,723	30.9%	2,142	19.1%
Felt unsafe at school	536	44.0%	528	35.2%	383	25.1%	384	31.5%	378	24.6%	265	20.8%	920	38.8%	906	30.8%	648	23.3%
Threatened on School Property	335	52.2%	332	39.8%	248	31.5%	428	32.2%	424	27.6%	318	20.8%	763	41.0%	756	32.9%	566	25.4%
Currently smoke cigarettes	254	55.5%	250	48.0%	199	37.7%	344	29.4%	341	23.8%	248	16.9%	598	40.5%	591	34.0%	447	26.2%
Currently using marijuana	1,168	39.8%	1,162	33.0%	913	21.8%	1,154	21.1%	1,149	16.4%	875	9.7%	2,322	30.5%	2,311	24.7%	1,788	15.9%
Current binge drinking	774	35.9%	770	29.2%	585	16.6%	630	19.4%	627	16.9%	468	10.9%	1,404	28.5%	1,397	23.7%	1,053	14.1%
Opiate use in past 30 days	350	50.0%	346	43.1%	307	30.3%	256	35.9%	255	35.3%	210	31.0%	606	44.1%	601	39.8%	517	30.6%
Illicit drugs 3 or more times	464	54.3%	457	45.1%	341	33.7%	473	34.7%	466	29.8%	322	24.5%	937	44.4%	923	37.4%	663	29.3%
Sleeping 5 or fewer hours nightly	1,422	37.6%	1,408	31.4%	1,100	18.8%	1,269	21.4%	1,258	19.2%	895	11.2%	2,691	30.0%	2,666	25.7%	1,995	15.4%
Low grades (Ds and Fs)	173	46.8%	173	45.1%	126	28.6%	318	27.7%	313	26.8%	223	17.9%	491	34.4%	486	33.3%	349	21.8%
Depression in past year	2,659	46.4%	2,652	37.5%	2,095	22.0%	1,459	39.3%	1,453	31.8%	1,152	17.3%	4,118	43.9%	4,105	35.5%	3,247	20.3%
Concussion	753	31.2%	745	24.8%	517	16.8%	930	19.4%	924	16.7%	621	13.4%	1,683	24.7%	1,669	20.3%	1,138	14.9%
No adversity or depression	1,477	3.7%	1,473	3.3%	1,335	0.6%	1,837	2.3%	1,835	2.0%	1,584	0.4%	3,314	2.9%	3,308	2.5%	2,919	0.5%

*Columns labeled n reflect the subgroup sample size that responded to the question on suicidal ideation, planning, or attempt and columns labeled % reflect the percent of that subgroup who endorsed suicidal ideation, planning, or making a suicide attempt. See Table 1 for definitions of these variables. Those who were classified as having no adversity and not being depressed (final row) said “no” to the following questions: felt unsafe at school; threatened on school property; QN17; ever forced to have sexual intercourse; forced sexual things in past year; forced sexual things while dating (if the person dated in the past year); QN22 (or missing); bullied on school property; bullied electronically; and being depressed in the past year*.

In the total sample, 12.8% reported sustaining a concussion in the past year (11.2% of girls and 14.4% of boys). Students who reported sustaining a concussion in the past year endorsed suicidal ideation more frequently (24.7%) than students who did not sustain a concussion in the past year [18.3%; χ^2^(1) = 36.69, *p* < 0.001; OR = 1.46 (1.29, 1.65)]. Students who reported sustaining a concussion endorsed a plan for suicide more frequently (20.3%) than students who did not sustain a concussion in the past year [15.0%; χ^2^(1) = 30.66, *p* < 0.001; OR = 1.45 (1.27, 1.65)]. Students who reported sustaining a concussion endorsed a suicide attempt more frequently (14.9%) than students who did not sustain a concussion in the past year [7.9%; χ^2^(1) = 61.72, *p* < 0.001; OR = 2.06 (1.72, 2.48)].

Multivariable binary logistic regression was used to examine the association between sustaining a concussion and suicidality after controlling for other known risk factors and correlates of suicidality. The results of the unadjusted and multivariable adjusted analyses for the girls are presented in Table 3 and for boys in Table 4. For girls, there was no significant association between sustaining a concussion and suicidal ideation, plans, or attempts after controlling for other risk factors. For boys, there was no significant association between sustaining a concussion and suicidal ideation or plans after controlling for other risk factors. However, there was a significant independent association between sustaining a concussion and suicide attempts.

**Table 3:** Logistic regressions predicting suicidality in girls.

**Suicidal ideation**											
	***B***	***SE***	**Wald**	***df***	***p***	**Adjusted odds ratio**	**95% Confidence interval**		**Unadjusted odds ratio**	**95% Confidence interval**	
							**Lower**	**Upper**		**Lower**	**Upper**
Physically Active 5 + days	−0.15	0.10	2.33	1	0.127	0.86	0.71	1.04	0.66	0.58	0.75
Sexual abuse/assault	0.68	0.14	24.99	1	**<0.001**	**1.97**	1.51	2.56	4.46	3.68	5.40
Felt unsafe at school	−0.01	0.15	0.01	1	0.933	0.99	0.74	1.32	2.68	2.23	3.22
Threatened at school	0.39	0.18	4.75	1	**0.029**	**1.47**	1.04	2.09	3.65	2.92	4.57
Bullied	0.59	0.10	37.49	1	**<0.001**	**1.80**	1.49	2.18	3.29	2.90	3.73
Low grades	0.31	0.25	1.54	1	0.215	1.37	0.83	2.24	2.84	2.09	3.85
Insufficient sleep	0.36	0.10	13.47	1	**<0.001**	**1.44**	1.18	1.74	2.34	2.05	2.67
Current binge drinking	0.14	0.13	1.30	1	0.254	1.15	0.90	1.48	1.94	1.65	2.28
Cigarettes (Current)	0.47	0.22	4.69	1	**0.030**	**1.60**	1.05	2.46	4.16	3.22	5.37
Illicit drugs	0.88	0.16	31.63	1	**<0.001**	**2.40**	1.77	3.25	4.13	3.40	5.02
Depression	2.38	0.12	428.60	1	**<0.001**	**10.84**	8.65	13.58	13.36	11.32	15.76
Concussion history	0.11	0.13	0.64	1	0.423	1.11	0.86	1.44	1.46	1.24	1.73
χ^2^(12) = 1,101.09, *p* < 0.001, Cox & Snell *R^2^* = 0.258, Nagelkerke *R^2^* = 0.385.											
**Suicide plan**											
Physically Active 5 + days	−0.02	0.10	0.04	1	0.837	0.98	0.80	1.19	0.72	0.63	0.83
Sexual abuse/assault	0.80	0.13	36.20	1	**<0.001**	**2.24**	1.72	2.91	4.61	3.80	5.60
Felt unsafe at school	−0.08	0.15	0.25	1	0.619	0.93	0.69	1.25	2.41	1.99	2.91
Threatened at school	0.11	0.18	0.34	1	0.558	1.11	0.78	1.58	2.86	2.27	3.60
Bullied	0.60	0.10	36.83	1	**<0.001**	**1.83**	1.51	2.23	3.27	2.86	3.74
Low grades	0.63	0.25	6.40	1	**0.011**	**1.88**	1.15	3.06	3.55	2.61	4.82
Insufficient sleep	0.43	0.10	18.22	1	**<0.001**	**1.54**	1.26	1.88	2.36	2.06	2.71
Current binge drinking	−0.02	0.13	0.03	1	0.860	0.98	0.76	1.26	1.86	1.57	2.21
Cigarettes (Current)	0.49	0.22	5.15	1	**0.023**	**1.63**	1.07	2.49	4.04	3.12	5.22
Illicit drugs	0.87	0.15	32.30	1	**<0.001**	**2.38**	1.76	3.20	3.75	3.07	4.57
Depression	2.18	0.12	309.52	1	**<0.001**	**8.83**	6.93	11.26	11.41	9.53	13.67
Concussion history	0.07	0.14	0.25	1	0.620	1.07	0.82	1.40	1.39	1.16	1.66
χ^2^(12) = 881.38, *p* < 0.001, Cox & Snell *R^2^* = 0.213, Nagelkerke *R^2^* = 0.337											
**Suicide attempt**											
Physically Active 5+ days	−0.08	0.14	0.38	1	0.539	0.92	0.71	1.20	0.54	0.42	0.70
Sexual abuse/assault	1.24	0.15	67.44	1	**<0.001**	**3.46**	2.57	4.65	6.54	5.22	8.19
Felt unsafe at school	−0.18	0.19	0.86	1	0.354	0.84	0.57	1.22	2.99	2.32	3.85
Threatened at school	0.26	0.21	1.55	1	0.213	1.30	0.86	1.97	5.38	3.94	7.34
Bullied	0.72	0.13	30.73	1	**<0.001**	**2.06**	1.60	2.67	3.73	3.08	4.52
Low grades	0.41	0.30	1.86	1	0.172	1.51	0.84	2.72	3.48	2.33	5.18
Insufficient sleep	0.32	0.13	5.87	1	**0.015**	**1.37**	1.06	1.78	2.37	1.95	2.87
Current binge drinking	−0.10	0.17	0.37	1	0.543	0.90	0.65	1.25	1.79	1.40	2.28
Cigarettes (Current)	0.41	0.25	2.75	1	0.097	1.51	0.93	2.47	5.48	4.05	7.43
Illicit drugs	0.90	0.17	27.02	1	**<0.001**	**2.46**	1.75	3.45	4.73	3.69	6.07
Depression	2.36	0.21	125.39	1	**<0.001**	**10.55**	6.98	15.93	14.93	10.85	20.54
Concussion history	0.05	0.18	0.09	1	0.768	1.05	0.74	1.49	1.70	1.32	2.19
χ^2^(12) = 570.75, *p* < 0.001. Cox & Snell *R^2^* = 0.156; Nagelkerke *R^2^* = 0.317											

*See Table 1 for definitions of these variables. Bold values indicate independently significant predictors in multivariable model*.

**Table 4:** Logistic regressions predicting suicidality in boys.

**Suicidal ideation**											
	***B***	***SE***	**Wald**	***df***	***p***	**Adjusted odds ratio**	**95% Confidence interval**		**Unadjusted odds ratio**	**95% Confidence interval**	
							**Lower**	**Upper**		**Lower**	**Upper**
Physically Active 5+ days	−0.26	0.12	4.65	1	**0.031**	**0.77**	0.61	0.98	0.58	0.49	0.67
Sexual abuse/assault	0.90	0.27	11.41	1	**0.001**	**2.46**	1.46	4.15	5.79	4.17	8.04
Felt unsafe at school	0.18	0.20	0.75	1	0.387	1.19	0.80	1.77	3.30	2.62	4.16
Threatened at school	0.51	0.19	7.45	1	**0.006**	**1.66**	1.15	2.38	3.50	2.81	4.37
Bullied	0.61	0.13	21.83	1	**<0.001**	**1.84**	1.43	2.38	4.14	3.50	4.89
Low grades	0.37	0.21	3.20	1	0.074	1.45	0.96	2.19	2.59	2.00	3.36
Insufficient sleep	0.24	0.13	3.55	1	0.059	1.27	0.99	1.64	2.14	1.82	2.53
Current binge drinking	−0.15	0.19	0.65	1	0.422	0.86	0.60	1.24	1.72	1.39	2.14
Cigarettes (Current)	0.22	0.24	0.89	1	0.345	1.25	0.79	1.98	2.98	2.33	3.81
Illicit drugs	0.72	0.18	15.35	1	**<0.001**	**2.06**	1.44	2.96	4.05	3.28	5.00
Depression	2.46	0.13	356.97	1	**<0.001**	**11.73**	9.09	15.15	15.13	12.53	18.26
Concussion history	0.22	0.16	1.93	1	0.164	1.24	0.92	1.69	1.69	1.41	2.03
*χ^2^*(12) = 744.88, *p* < 0.001, Cox & Snell *R^2^* = 0.193, Nagelkerke *R^2^* = 0.358											
**Suicide plan**											
Physically active 5+ Days	−0.19	0.13	2.26	1	0.133	0.83	0.65	1.06	0.59	0.50	0.70
Sexual abuse/assault	1.25	0.26	22.48	1	**<0.001**	**3.49**	2.08	5.84	5.90	4.22	8.26
Felt unsafe at school	−0.26	0.22	1.38	1	0.240	0.77	0.50	1.19	2.83	2.20	3.64
Threatened at school	0.54	0.19	8.07	1	**0.004**	**1.71**	1.18	2.49	3.49	2.77	4.40
Bullied	0.45	0.14	10.63	1	**0.001**	**1.57**	1.20	2.06	3.76	3.14	4.49
Low grades	0.48	0.21	5.22	1	**0.022**	**1.62**	1.07	2.46	3.15	2.41	4.11
Insufficient sleep	0.38	0.13	8.15	1	**0.004**	**1.46**	1.13	1.89	2.43	2.04	2.90
Current binge drinking	−0.03	0.19	0.03	1	0.874	0.97	0.67	1.41	1.77	1.40	2.22
Cigarettes (Current)	0.08	0.24	0.11	1	0.744	1.08	0.67	1.75	2.70	2.07	3.52
Illicit drugs	0.81	0.19	18.51	1	**<0.001**	**2.24**	1.55	3.24	3.89	3.12	4.85
Depression	2.37	0.14	292.43	1	**<0.001**	**10.75**	8.19	14.11	11.94	9.80	14.54
Concussion history	0.12	0.16	0.51	1	0.474	1.12	0.82	1.55	1.76	1.44	2.14
*χ^2^*(12) = 629.70, *p* < 0.001, Cox & Snell *R^2^* = 0.166, Nagelkerke *R^2^* = 0.330											
**Suicidal attempt**											
	***B***	***SE***	**Wald**	***df***	***p***	**Adjusted odds ratio**	**95% Confidence interval**		**Unadjusted odds ratio**	**95% Confidence interval**	
							**Lower**	**Upper**		**Lower**	**Upper**
Physically Active 5 + days	−0.29	0.19	2.39	1	0.122	0.74	0.51	1.08	0.54	0.42	0.70
Sexual abuse/assault	1.24	0.31	16.20	1	**<0.001**	**3.47**	1.89	6.36	9.49	6.43	14.00
Felt unsafe at school	0.15	0.28	0.29	1	0.592	1.16	0.67	2.03	5.00	3.59	6.97
Threatened at school	0.51	0.25	4.03	1	**0.045**	**1.66**	1.01	2.74	5.38	3.94	7.34
Bullied	0.55	0.20	7.93	1	**0.005**	**1.74**	1.18	2.56	4.52	3.47	5.88
Low grades	0.74	0.28	7.05	1	**0.008**	**2.10**	1.21	3.62	4.08	2.81	5.92
Insufficient sleep	0.25	0.19	1.65	1	0.199	1.28	0.88	1.88	2.68	2.05	3.50
Current binge drinking	0.00	0.27	0.00	1	0.987	1.00	0.59	1.71	2.45	1.76	3.41
Cigarettes (Current)	0.09	0.33	0.07	1	0.791	1.09	0.57	2.10	4.09	2.84	5.88
Illicit drugs	0.95	0.25	14.54	1	**<0.001**	**2.59**	1.59	4.23	7.06	5.22	9.56
Depression	2.83	0.28	104.46	1	**<0.001**	**16.92**	9.83	29.10	13.91	9.93	19.50
Concussion history	0.68	0.22	9.62	1	**0.002**	**1.98**	1.28	3.04	3.13	2.37	4.15
*χ^2^*(12) = 378.51, *p* < 0.001, Cox & Snell *R^2^* = 0.116, Nagelkerke *R^2^* = 0.351											

*See Table 1 for definitions of these variables. Bold values indicate independently significant predictors in multivariable model*.

Exploratory logistic regressions were conducted without including depression as a variable, given its strong association with suicidality, as both a direct determinant and a mediator variable between the other covariates, and also how it might arise as a consequence of concussion. In addition, other variables were removed to examine whether concussion was associated with suicidality after removing additional variance associated with those variables, and to reduce the large number of predictors from 12 to 8 (i.e., Tables 5, 6). Feeling unsafe at school was removed because it was not a significant predictor in the larger model and due to some conceptual redundancy (shared variance) with bullying. Being threatened at school was also removed due to some potential redundancy with bullying. Current cigarette use was removed because it was a low frequency behavior relating to substance use (Table 2), and we wanted to keep higher frequency substance use behaviors such as binge drinking and illicit substance use in the model.

**Table 5:** Logistic regressions predicting suicidality in girls (reduced set of predictors).

**Suicidal ideation**											
	***B***	***SE***	**Wald**	***df***	***p***	**Adjusted odds ratio**	**95% Confidence interval**		**Unadjusted odds ratio**	**95% Confidence interval**	
							**Lower**	**Upper**		**Lower**	**Upper**
Physically Active 5 + days	−0.31	0.09	12.78	1	**<0.001**	**0.73**	0.62	0.87	0.66	0.58	0.75
Sexual abuse/assault	1.01	0.12	69.45	1	**<0.001**	**2.75**	2.17	3.50	4.46	3.68	5.40
Bullied	1.06	0.08	156.94	1	**<0.001**	**2.89**	2.45	3.41	3.29	2.90	3.73
Low grades	0.46	0.23	4.14	1	**0.042**	**1.59**	1.02	2.47	2.84	2.09	3.85
Insufficient sleep	0.69	0.09	59.46	1	**<0.001**	**1.99**	1.67	2.37	2.34	2.05	2.67
Current binge drinking	0.38	0.11	11.54	1	**0.001**	**1.46**	1.18	1.83	1.94	1.65	2.28
Illicit drugs	1.17	0.14	70.85	1	**<0.001**	**3.22**	2.45	4.23	4.13	3.40	5.02
Concussion history	0.14	0.12	1.41	1	0.235	1.15	0.91	1.46	1.46	1.24	1.73
χ^2^(8) = 523.96, *p* < 0.001, Cox & Snell *R^2^* = 0.130, Nagelkerke *R^2^* = 0.194											
**Suicide plan**											
Physically Active 5+ Days	−0.18	0.09	3.78	1	0.052	0.83	0.69	1.00	0.72	0.63	0.83
Sexual abuse/assault	1.05	0.12	72.52	1	**<0.001**	**2.85**	2.24	3.63	2.86	2.27	3.60
Bullied	1.00	0.09	124.58	1	**<0.001**	**2.73**	2.29	3.25	3.27	2.86	3.74
Low grades	0.69	0.23	9.14	1	**0.002**	**1.99**	1.27	3.11	3.55	2.61	4.82
Insufficient sleep	0.71	0.09	57.31	1	**<0.001**	**2.04**	1.69	2.45	2.36	2.06	2.71
Current binge drinking	0.25	0.12	4.51	1	**0.034**	**1.29**	1.02	1.63	1.86	1.57	2.21
Illicit drugs	1.16	0.14	68.63	1	**<0.001**	**3.19**	2.43	4.20	3.75	3.07	4.57
Concussion history	0.12	0.13	0.91	1	0.341	1.13	0.88	1.45	1.39	1.16	1.66
χ^2^(8) = 464.32, *p* < 0.001, Cox & Snell *R^2^* = 0.116, Nagelkerke *R^2^* = 0.184											
**Suicide attempt**											
Physically Active 5+ days	−0.20	0.13	2.40	1	0.121	0.82	0.64	1.05	0.54	0.42	0.70
Sexual abuse/assault	1.43	0.14	100.31	1	**<0.001**	**4.20**	3.17	5.56	6.54	5.22	8.19
Bullied	1.10	0.12	82.15	1	**<0.001**	**3.01**	2.37	3.82	3.73	3.08	4.52
Low grades	0.54	0.29	3.44	1	0.064	1.71	0.97	3.03	3.48	2.33	5.18
Insufficient sleep	0.57	0.13	20.12	1	**<0.001**	**1.76**	1.37	2.25	2.37	1.95	2.87
Current binge drinking	0.08	0.16	0.25	1	0.614	1.08	0.79	1.48	1.79	1.40	2.28
Illicit drugs	1.21	0.16	53.48	1	**<0.001**	**3.34**	2.42	4.62	4.73	3.69	6.07
Concussion history	0.11	0.17	0.38	1	0.537	1.11	0.79	1.55	1.70	1.32	2.19
χ^2^(8) = 361.54, *p* < 0.001, Cox & Snell *R^2^* = 0.100, Nagelkerke *R^2^* = 0.203											

*See Table 1 for definitions of these variables. Bold values indicate independently significant predictors in multivariable model*.

**Table 6:** Logistic regressions predicting suicidality in boys (reduced set of predictors).

**Suicidal ideation**											
	***B***	***SE***	**Wald**	***df***	***p***	**Adjusted odds ratio**	**95% Confidence interval**		**Unadjusted odds ratio**	**95% Confidence interval**	
							**Lower**	**Upper**		**Lower**	**Upper**
Physically Active 5 + days	−0.43	0.11	16.46	1	**<0.001**	**0.65**	0.53	0.80	0.58	0.49	0.67
Sexual abuse/assault	1.22	0.23	28.15	1	**<0.001**	**3.38**	2.16	5.31	5.79	4.17	8.04
Bullied	1.27	0.11	129.54	1	**<0.001**	**3.56**	2.86	4.43	4.14	3.50	4.89
Low grades	0.54	0.18	8.66	1	**0.003**	**1.71**	1.20	2.45	2.59	2.00	3.36
Insufficient sleep	0.51	0.12	19.93	1	**<0.001**	**1.67**	1.33	2.09	2.14	1.82	2.53
Current binge drinking	0.02	0.16	0.01	1	0.920	1.02	0.74	1.39	1.72	1.39	2.14
Illicit drugs	0.94	0.16	35.11	1	**<0.001**	**2.55**	1.87	3.48	4.05	3.28	5.00
Concussion history	0.30	0.14	4.65	1	**0.031**	**1.35**	1.03	1.77	1.69	1.41	2.03
χ^2^(8) = 305.61, *p* < 0.001, Cox & Snell *R^2^* = 0 082, Nagelkerke *R^2^* = 0.152											
**Suicide plan**											
Physically Active 5+ days	−0.34	0.11	9.09	1	**0.003**	**0.71**	0.57	0.89	0.59	0.50	0.70
Sexual abuse/assault	1.45	0.23	39.63	1	**<0.001**	**4.25**	2.71	6.67	5.90	4.22	8.26
Bullied	1.08	0.12	80.98	1	**<0.001**	**2.95**	2.33	3.73	3.76	3.14	4.49
Low grades	0.66	0.19	12.26	1	**<0.001**	**1.93**	1.34	2.79	3.15	2.41	4.11
Insufficient sleep	0.62	0.12	26.49	1	**<0.001**	**1.86**	1.47	2.36	2.43	2.04	2.90
Current binge drinking	0.07	0.17	0.20	1	0.656	1.08	0.78	1.49	1.77	1.40	2.22
Illicit drugs	1.03	0.16	40.53	1	**<0.001**	**2.81**	2.05	3.87	3.89	3.12	4.85
Concussion history	0.23	0.15	2.46	1	0.117	1.26	0.94	1.68	1.76	1.44	2.14
χ^2^(8) = 278.91, *p* < 0.001, Cox & Snell *R^2^* = 0.075, Nagelkerke *R^2^* = 0.149											
**Suicide attempt**											
Physically Active 5 + days	−0.47	0.17	7.08	1	**0.008**	**0.63**	0.45	0.88	0.54	0.42	0.70
Sexual abuse/assault	1.60	0.28	31.95	1	**<0.001**	**4.95**	2.84	8.62	9.49	6.43	14.00
Bullied	1.29	0.17	55.50	1	**<0.001**	**3.65**	2.60	5.13	4.52	3.47	5.88
Low grades	1.03	0.25	16.63	1	**<0.001**	**2.80**	1.71	4.60	4.08	2.81	5.92
Insufficient Sleep	0.50	0.18	7.45	1	**0.006**	**1.64**	1.15	2.34	2.68	2.05	3.50
Current binge drinking	0.02	0.24	0.01	1	0.936	1.02	0.64	1.63	2.45	1.76	3.41
Illicit drugs	1.21	0.22	30.46	1	**<0.001**	**3.34**	2.18	5.13	7.06	5.22	9.56
Concussion history	0.82	0.20	17.04	1	**<0.001**	**2.27**	1.54	3.34	3.13	2.37	4.15
χ^2^(8) = 228.73, *p* < 0.001, Cox & Snell *R^2^* = 0.070, Nagelkerke *R^2^* = 0.206											

*See Table 1 for definitions of these variables. Bold values indicate independently significant predictors in multivariable model*.

Again, for girls, there was no significant association between sustaining a concussion and suicidal ideation, plans, or attempts after controlling for other risk factors. For boys, there was a modest association between sustaining a concussion and suicidal ideation with the reduced set of predictors that was not observed when including the larger set of predictors in the model [*p* = 0.031, OR = 1.35 (95% CI: 1.03, 1.77)], meaning variance in suicidal ideation was explained by the removed predictors (e.g., current cigarette use and feeling unsafe or being threatened at school). The significant independent association between sustaining a concussion and suicide attempts [*p* < 0.001, OR = 2.27 (1.54, 3.34)] was observed in the reduced model, as was observed in the model with the full set of predictors. There was no significant association between sustaining a concussion and suicide planning after controlling for other risk factors.

## Discussion

There was an association between sustaining a concussion in sports or recreational activity and suicidality in high school students surveyed in the United States in 2019. This is consistent with prior research using epidemiological survey data collected during 2013 (45) and 2017 (41–43, 46). After controlling for other variables, however, the associations between concussion and suicidality were greatly attenuated or became non-significant. For high school girls, there were no statistically significant associations in the adjusted analysis between concussion and suicidal thoughts, plans, or attempts. For girls, significant predictors of suicidal thoughts, in decreasing order of magnitude, were depression, sexual abuse/assault, current cigarette use, lifetime illicit drug use, being threatened at school, being bullied at school or electronically, low grades, feeling unsafe at school, insufficient sleep, and current binge drinking. For high school boys, there were no statistically significant associations in the adjusted analysis between concussion and suicidal thoughts or plans, but there was an association between concussion and suicide attempts. For boys, significant predictors of suicidal thoughts, in decreasing order of magnitude, were depression, sexual abuse/assault, being bullied at school or electronically, lifetime illicit drug use, being threatened at school, feeling unsafe at school, current cigarette use, low grades, insufficient sleep, and current binge drinking. Being physically active in the last 5 days was protective against suicidal ideation in both boys and girls, reducing the odds of suicidal thoughts by about half.

Past researchers using data from the YRBS have reported that suicidality in high school students is associated with female sex (2, 4), sexual minority status (47–49), adverse childhood experiences (50, 51), physical teen dating violence (52), forced sexual intercourse (18, 53), body weight perceptions [both overweight (54, 55) and underweight (55)], illicit drug use (13, 18), prescription opioid misuse (56–58) [especially in girls (59)], alcohol use (15, 18, 60, 61), marijuana use (13, 15, 62), bullying (18, 55, 63, 64) and cyberbullying (18, 55, 63–65), frequent physical fighting (66), excessive television/video game/internet use (67, 68), insufficient sleep (69), and even soft drink consumption (70). With so many variables being associated with suicidality in youth—especially given the very large national sample allowing the identification of statistically significant findings with small effect sizes—it is not particularly surprising that concussion was also associated in the present study, in univariate analyses, and in prior studies using the 2013 (45) and 2017 YRBS (41–43, 46). These studies found associations between concussion and suicidal ideation (41, 42, 45), planning (41, 45), and attempts (41–43, 45) through unadjusted analyses and multivariable analyses adjusting for some relevant variables. These prior studies controlled for demographic variables (e.g., age, gender, race/ethnicity, sexual orientation) and some other variables associated with mental health problems, such as alcohol use (45); bullying (42, 46); grades (41, 43); and physical activity, grades, sports participation, poor sleep, alcohol use, marijuana use, and depression (41). In general, the previous studies controlled for a relatively small number of variables, however, and none controlled for a history of sexual abuse or assault. The current study included all of these variables and more in a single multivariable logistic regression model, observing only an increased risk of suicide attempt in boys associated with concussion. When including multiple variables in the model, most predictors have a large reduction in effect size because the predictors are interrelated (i.e., they share variance), and the predictors with the smallest univariate odd ratios (including concussion) become non-significant (see Tables 3, 4).

As prior authors using YRBS data have noted, we cannot infer a causal association between concussion and suicidality given this is a cross-sectional survey study (41–43, 45, 46). That said, the association may be due, at least in part, to concussion being a major life stressor in boys, especially those with high athletic identity in their sport. Sustaining an injury in sports can be a major life stressor, and it can trigger or unmask difficulties with depression and suicidality (71). Concussions can remove athletes from their peer group and regular physical exercise, disrupt their academic performances, and lead to uncertainty about their futures. Such a disruption may have substantial psychological consequences for some adolescent boys. Among samples of predominantly male athletes, greater athletic identity has been associated with greater symptoms of depression following sport-related injuries (72), which may explain, in part, the current finding.

The array of vulnerability and risk factors for suicidality likely co-occur as precipitants to, and/or consequences of, psychological distress. Adolescents who engage in substance use may do so to cope with underlying emotional problems, or they may do so as recreation with their peers, with increased or riskier use leading to psychological distress. Adolescents who have experienced sexual violence may reside in environments with greater risk for being bullied, threatened, or further victimized, contributing to mental health problems and increasing risk for suicidality. A student-athlete who experiences a concussion may be removed from the social support of a team setting and may exercise less frequently when removed from practice or play. Considering the relationship between physical activity, mental health, and cognitive functioning (73), student-athletes removed from sport may have worse mental health, struggle more in school, and engage in high-risk activities to cope with their distress (e.g., substance use), which may contribute to greater distress.

### Limitations

This study has several important limitations. The methodology, of course, was a survey—and this cross-sectional survey study does not allow us to draw causal inferences. The survey was administered during a single class period and the students were surrounded by their peers. The CDC implemented a system check that attempted to identify surveys that reflected mischievous responding by excluding those who responded the same way 15 or more times in a row. There are other possible response biases and styles that could have been operative among some students, such as social desirability, under or over-reporting, extreme response styles, acquiescence response styles, or yea-saying and nay-saying. Attempting to study different types of response styles and response biases is beyond the scope of this study; it is possible that some students' survey responses were influenced by certain bias, styles, or mischievous responding. The students were not asked any details about their concussions, such as the severity of their symptoms, the extent to which they received treatment or rehabilitation, or their recovery time—and there is no way to determine, from this cross-sectional survey, how many students developed depression and/or suicidality after sustaining a concussion, or whether depression or suicidality appeared to be causally related to sustaining a concussion.

There are also important statistical considerations. It is important to appreciate that we *did not* attempt to Model suicidality in youth, *per se*, but rather we aimed to examine the association of concussion with suicidality while considering multiple other variables of interest. There would be, as expected, some modest overfitting of the statistical model given the large number of variables included, although this overfitting is mitigated by the very large sample size. However, the most notable statistical issue relates to confounding and mediation. Depression is by far the strongest correlate with suicidality (i.e., greatest effect size). However, several of the other variables, for example sexual abuse/assault, illicit drug use, and bullying, are determinants of, or presumably causally linked to, depression. Thus, depression is a likely mediator between the predictors and suicidality. By including depression in the statistical model there is an introduction of bias due to either confounding (i.e., bidirectional relationship between depression and covariates) or mediation (i.e., a directional relationship). Therefore, the models are also presented without including depression (Tables 5, 6). It is also important to appreciate that other variables might contribute to confounding, mediation, or both. For example, bullying might also be linked to substance use. These interrelationships contribute to the large reductions in effect sizes, in the analyses, between the unadjusted (single variables) and adjusted (multiple variables) effect sizes.

## Conclusions

Suicidality in high school students is a public health problem. There are many known predictors and correlates of suicidality in youth, with depression having, by far, the strongest association with suicidality in the present study. Other significant predictors, in both boys and girls, included a history of sexual abuse or assault and the use of illicit drugs. Concussion was not a significant predictor of ideation, plans, or attempts in girls after controlling for other variables, and it was not a significant predictor of ideation or plans in boys in multivariable analyses. Concussion remained a significant predictor in boys for suicide attempts after controlling for other variables. That said, children and adolescents presenting to specialty clinics for treatment and rehabilitation following concussion often experience emotional health difficulties, such as anxiety and depression (74, 75). Having pre-injury mental health problems is a risk factor for experiencing post-injury anxiety and depression (74, 76, 77)—and these are risk factors for suicidality. Because concussion can disrupt a young person's lifestyle in fairly dramatic ways, there are multiple potential reasons why some students who sustain concussions might evolve into experiencing depression and suicidality, and health care providers are encouraged to be mindful of this possibility and to provide swift, evidence-informed mental health care for youth who are suffering.

## Data Availability Statement

Publicly available datasets were analyzed in this study. This data can be found here: Centers for Disease Control and Prevention (CDC) Youth Risk Behavior Surveillance System (YRBSS), https://www.cdc.gov/healthyyouth/data/yrbs/data.htm.

## Ethics Statement

The Institutional Review Board of the CDC approved the protocol for the YRBS. Survey procedures were designed to protect students' privacy by allowing for anonymous and voluntary participation. Before survey administration, local parental permission procedures were followed.

## Author Contributions

GI: conceptualized the study, conducted the literature review, conceptualized the statistical analyses, conducted the analyses, and drafted portions of the manuscript. JK: assisted with the literature review, conducted the analyses, drafted portions of the manuscript, and reviewed and approved the final manuscript. All authors contributed to the article and approved the submitted version.

## Funding

GI has received unrestricted philanthropic support from ImPACT Applications, Inc., the Mooney-Reed Charitable Foundation, the National Rugby League, the Boston Bolts, and the Spaulding Research Institute. None of the above entities were involved in the study design, collection, analysis, interpretation of data, the writing of this article, or the decision to submit it for publication.

## Conflict of Interest

GI serves as a scientific advisor for NanoDx^®^ Inc., Sway Operations, LLC, and Highmark, Inc. He has a clinical and consulting practice in forensic neuropsychology, including expert testimony, involving individuals who have sustained mild TBIs (including athletes). He has received research funding from several test publishing companies, including ImPACT Applications, Inc., CNS Vital Signs, and Psychological Assessment Resources (PAR, Inc.). He has received research funding as a principal investigator from the National Football League, and salary support as a collaborator from the Harvard Integrated Program to Protect and Improve the Health of National Football League Players Association Members. The remaining author declares that the research was conducted in the absence of any commercial or financial relationships that could be construed as a potential conflict of interest.

## Publisher's Note

All claims expressed in this article are solely those of the authors and do not necessarily represent those of their affiliated organizations, or those of the publisher, the editors and the reviewers. Any product that may be evaluated in this article, or claim that may be made by its manufacturer, is not guaranteed or endorsed by the publisher.
